# Dietary Glycemic Index, Glycemic Load, and Risk of Coronary Heart Disease, Stroke, and Stroke Mortality: A Systematic Review with Meta-Analysis

**DOI:** 10.1371/journal.pone.0052182

**Published:** 2012-12-20

**Authors:** Jingyao Fan, Yiqing Song, Yuyao Wang, Rutai Hui, Weili Zhang

**Affiliations:** 1 Sino-German Laboratory for Molecular Medicine, the State Key Laboratory of Cardiovascular Diseases, FuWai Hospital, National Center for Cardiovascular Diseases, Peking Union Medical College and Chinese Academy of Medical Sciences, Beijing, People’s Republic of China; 2 Institute of Vascular Medicine, Peking University Third Hospital, Ministry of Health, Beijing, People’s Republic of China; 3 Department of Medicine, Division of Preventive Medicine, Brigham and Women's Hospital, Harvard Medical School, Boston, Massachusetts, United States of America; Virginia Commenwealth University, United States of America

## Abstract

**Background:**

The relationship between dietary glycemic index, glycemic load and risk of coronary heart disease (CHD), stroke, and stroke-related mortality is inconsistent.

**Methods:**

We systematically searched the MEDLINE, EMBASE, and Science Citation Index Expanded databases using glycemic index, glycemic load, and cardiovascular disease and reference lists of retrieved articles up to April 30, 2012. We included prospective studies with glycemic index and glycemic load as the exposure and incidence of fatal and nonfatal CHD, stroke, and stroke-related mortality as the outcome variable. Pooled relative risks (RR) and 95% confidence intervals (CI) were calculated using random-effects models.

**Results:**

Fifteen prospective studies with a total of 438,073 participants and 9,424 CHD cases, 2,123 stroke cases, and 342 deaths from stroke were included in the meta-analysis. Gender significantly modified the effects of glycemic index and glycemic load on CHD risk, and high glycemic load level was associated with higher risk of CHD in women (RR = 1.49, 95%CI 1.27−1.73), but not in men (RR = 1.08, 95%CI 0.91−1.27). Stratified meta-analysis by body mass index indicated that among overweight and obese subjects, dietary glycemic load level were associated with increased risk of CHD (RR = 1.49, 95%CI 1.27−1.76; *P* for interaction = 0.003). Higher dietary glycemic load, but not glycemic index, was positively associated with stroke (RR = 1.19, 95% CI 1.00−1.43). There is a linear dose-response relationship between dietary glycemic load and increased risk of CHD, with pooled RR of 1.05 (95%CI 1.02−1.08) per 50-unit increment in glycemic load level.

**Conclusion:**

High dietary glycemic load is associated with a higher risk of CHD and stroke, and there is a linear dose-response relationship between glycemic load and CHD risk. Dietary glycemic index is slightly associated with risk of CHD, but not with stroke and stroke-related death. Further studies are needed to verify the effects of gender and body weight on cardiovascular diseases.

## Introduction

High carbohydrate intake has adverse effects on lipid and glucose metabolism [1]–[3], thereby creating potential worries to increase the risk of cardiovascular diseases [4]. Dietary carbohydrates vary in their ability to increase postprandial blood glucose levels depending on different chemical structures, particle sizes, fiber contents, and food processing. The glycemic index (GI) measure is thus an indicator of how quickly a carbohydrate can be absorbed as glucose compared with a reference, which is generally white bread or pure glucose [5], [6]. Because the amount of carbohydrate in a food can vary, the glycemic load (GL) measure is used to represent both quantity and quality of carbohydrates and calculated by multiplying the GI of a food item with its carbohydrate content.

Dietary GI and GL have increased in recent years because of increases in carbohydrate intake and changes in food processing, especially in the lower- and middle-income countries of the Asia-Pacific region [4]. High-GI and GL diets might lead to vessel dysfunction, an important pathogenesis of cardiovascular disease. In Japan, the incidence of hemorrhagic stroke declined in parallel with a decrease in carbohydrate intake and increased fat and protein intake [7]. In a study of Chinese Americans, participants who consumed a high-carbohydrate and low-fat diet had lower high density lipoprotein and total cholesterol concentrations compared with elderly Whites [8]. These characteristics were similar to those of urban populations in China, where hemorrhagic stroke is the major cause of cardiovascular disease [9]. Given that an alarming increase in the prevalence of cardiovascular diseases worldwide, insight into the role of specific dietary factors has public health importance for prevention strategies.

Accumulating epidemiological studies have suggested that high dietary GI and GL could be detrimental in regard to the risk of coronary heart disease [10]–[13], but the results are inconsistent in various populations [14]–[17]. A recent meta-analysis of prospective cohort studies showed that individuals with the highest level of dietary GL and GI have approximately 1.3-fold increased risk of coronary heart disease in women but not in men compared with those with the lowest level [18]. Since that review was published, new evidence is available [19]–[22]. In addition, the influence of body weight on the relations of dietary GL and GI to CHD risk was reported positive in some studies [11], [13], [16], but nonsignificant in others [12], [15]. Several recent studies have also published data suggesting that high dietary GI and GL contribute to the risk of stroke and stroke-related mortality, but there has been no systematic evaluation of these inconclusive findings [11], [15], [22], [23]–[25]. To date, no randomized trials have directly assessed the effects of low GL or GI diets on the end-points of cardiovascular diseases; however, short-term intervention studies have indicated beneficial effects of low GL or GI diets on unfavorable cardiovascular risk profile [26]–[30]. Hence, the purpose of the current study was to update the previous meta-analysis of the association between dietary GL, GI and risk of CHD and to conduct a systematical assessment of the evidence on the risk of stroke and stroke-related mortality.

## Methods

### Search Strategy

We searched for all published prospective studies that described the associations between GL, GI and the risk of incident CHD, stroke, and stroke-related mortality. A systematic literature search was performed using the MEDLINE (Pubmed) and EMBASE databases and was supplemented through the manual review of reference list of obtained articles up to April 30, 2012. The following terms were used: ((“glycaemic index”[All Fields] OR “glycemic index”[MeSH Terms] OR (“glycemic”[All Fields] AND “index”[All Fields]) OR “glycemic index”[All Fields]) OR (“glycemic”[All Fields] AND “load”[All Fields])) AND ((“coronary disease”[MeSH Terms] OR (“coronary”[All Fields] AND “disease”[All Fields]) OR “coronary disease”[All Fields] OR (“coronary”[All Fields] AND “heart”[All Fields] AND “disease”[All Fields]) OR “coronary heart disease”[All Fields] OR “coronary artery disease”[MeSH Terms] OR (“coronary”[All Fields] AND “artery”[All Fields] AND “disease”[All Fields]) OR “coronary artery disease”[All Fields] OR (“coronary”[All Fields] AND “heart”[All Fields] AND “disease”[All Fields])) OR (“stroke”[MeSH Terms] OR “stroke”[All Fields]) OR (“cardiovascular diseases”[MeSH Terms] OR (“cardiovascular”[All Fields] AND “diseases”[All Fields]) OR “cardiovascular diseases”[All Fields] OR (“cardiovascular”[All Fields] AND “disease”[All Fields]) OR “cardiovascular disease”[All Fields])). No language restriction was applied for searching and study inclusion. Our systematic review was conducted according to the Meta-analysis of Observational Studies in Epidemiology (MOOSE) guidelines [31].

### Eligibility Criteria

Studies were considered eligible for meta-analysis if they met the following criteria: the study had a prospective design; the exposure was dietary GL or GI; the outcome was incident CHD or stroke; and the study excluded participants with known pre-existing cardiovascular disease. Because the nested case-control study in a prospective cohort is just an efficient sampling of the same cohort study and thus retains the same prospective advantages of the cohort, and dietary information was collected among apparently healthy participants at baseline before the development of outcome of interest, the study by Pierucci et al [20] was included as a prospective study. We excluded literature reviews, cross-sectional studies, case-control studies, and animal studies.

### Data Extraction

Data extraction was conducted independently by 2 authors (J.F., Y.W.), using a standardized data extraction form. To resolve discrepancies, a third investigator (W.Z.) was consulted. We contacted authors of the original studies in the case of missing data. For each included article, study characteristics were recorded as follows: authors, publication year, country of origin, name of study, study design, features of study population (sample size, age, proportion of men, and mean body mass index [BMI]), duration of the follow-up, mean (standard deviation, SD) or median values for the GI or GL, reference food used for GI calculation, the criterion for ascertainment of outcomes, numbers of incident CHD or stroke cases, and confounding factors that were adjusted for in the multivariable analysis. Accepted standardized quality scores for observational studies are not available. Therefore, study’s quality was assessed by review of study design, including inclusion and exclusion criteria, assessment of exposure, assessment of outcome, control of confounding, and evidence of bias. Each of the 5 quality criteria was evaluated and scored on an integer scale (0 or 1, with 1 being better) and summed. Quality scores from 0 to 3 were considered lower quality and 4 to 5 higher quality.

In the original articles which used tertiles, quartiles, quintiles, deciles, or percentiles of GI and GL as categories for dietary GI and GL levels, we extracted median values, numbers of cases/noncases, relative risks (RRs), and 95% confidence intervals (CIs). For studies that reported several multivariable-adjusted RRs, we extracted the effect estimate that was most fully adjusted for potential confounders. If medians for categories of dietary GL and GI were not reported, approximate medians were estimated using the midpoint of the lower and upper bounds (or using the mean when the midpoint could not be estimated).

### Statistical Analysis

We used the multivariate-adjusted odds ratio and hazard ratio reported in the original articles, and the odds ratios in the nested case-control study design were assumed to be accurate estimates of risk ratio. We therefore consider these estimates as relative risks.

In CHD risk-related analysis, records from the studies by Sieri et al [12] and Grau et al [17] were entered separately for men and women, because only gender-specific RRs were presented for these 2 studies. A total of 12 separate estimates from 10 studies [10]–[17], [19], [20] were included in the analysis for the association between categories of GI and GL and CHD risk. In addition, 3 studies reported results for continuous GI and GL levels [14], [21], [22].

In stroke risk-related analysis, 3 studies [11], [15], [23] used category variable describing GI and GL levels, while 1 study [22] used continuous variable for GI and GL levels. Records from the studies by Levitan et al [15] and Oh et al [23] were entered separately for ischemic stroke and hemorrhagic Stroke. Thus, we included 5 separate estimates in the analysis of category levels of GL or GI and stroke risk. In stroke mortality analysis, records from the study by Oba et al [24] were entered for men and women separately. A total of 3 separate estimates from 2 studies [24], [25] were included in the analysis for the association between category levels of GI and stroke mortality.

Fixed- and random-effects models were used to calculate the pooled risk estimates and 95% CI by comparing the highest and lowest categories of exposure. In the fixed-effects model, the pooled RR was obtained by averaging the lnRRs weighted by the inverses of their variances. In the random-effects model, DerSimonian and Laird’s method was used to further incorporate between-study heterogeneity [32]. We reported the pooled risk estimates from the random-effects model if the test for heterogeneity was significant. The Cochran Q test and the *I* ^2^ statistics were used to examine statistical heterogeneity across studies. *I* ^2^ was calculated based on the formula *I* ^2^ = 100%×(Q–df)/Q.

In the secondary analysis, we estimated the dose-response relationship based on available data for categories of dietary GL or GI on median dose, number of cases and participants, and effect estimates with corresponding standard errors using the generalized least-squares trend estimation (GLST) analysis [33]. We used the 2-stage GLST method because this allowed us to combine the GLST-estimated study-specific slopes with the results from studies that only reported effect estimates for continuous associations. A quadratic term of dietary GL and GI was added in the analysis to test if the associations of the natural logarithm of RRs with increasing GL and GI were nonlinear; the changes in model fit were tested using the likelihood ratio test [33].

Potential publication bias was assessed by using the Egger’s regression test [34] and visual inspection of a funnel plot [35], dependent on the degree of heterogeneity observed. All tests were 2-sided and *P* value <0.05 was considered statistically significant. All analyses were performed using STATA 10.1 software (STATA Corp, College Station, Texas, USA).

## Results

**Figure 1:**
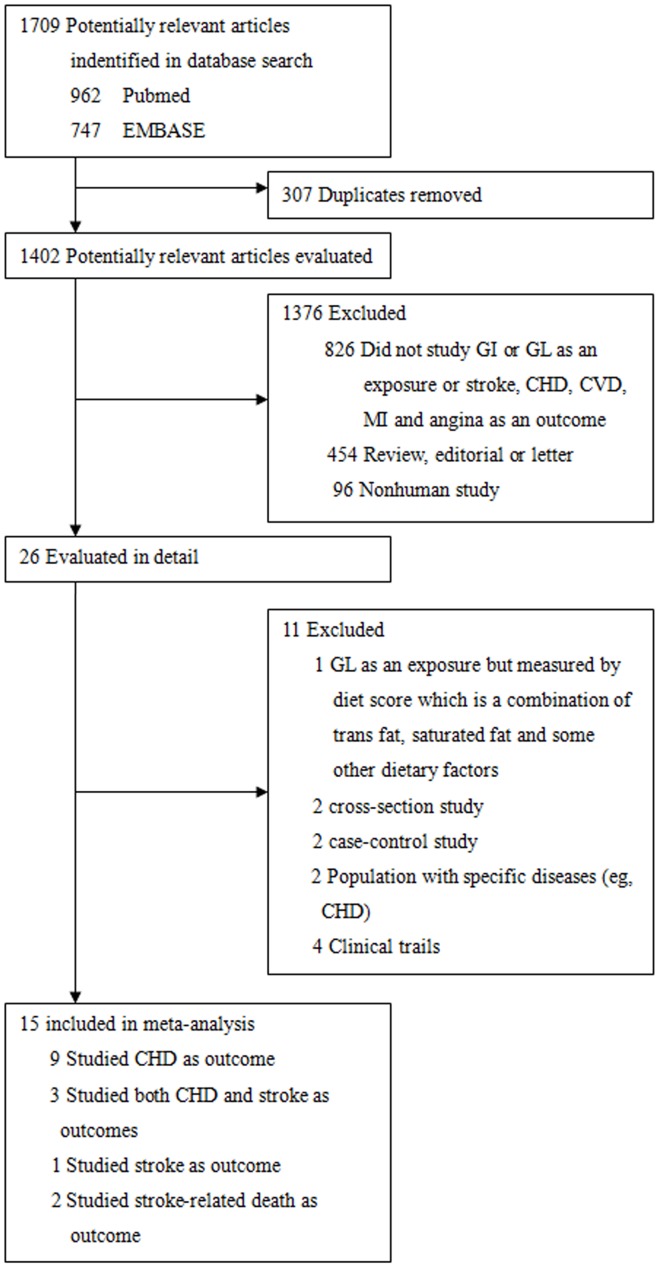
Selection of studies for meta-analysis. Literatures search was conducted to identify articles up to April 30, 2012. Abbreviation: GI, glycemic index; GL, glycemic load; CHD, coronary heart disease; MI, myocardial infraction; CVD, cardiovascular disease.

The results of the literature search are shown in Figure 1. We identified 15 prospective studies (9 studies used CHD as outcome, 3 used CHD and stroke as separate outcome, 1 used stroke as outcome, and 2 used stroke-related death as outcome), comprising 438,073 individuals in whom 9,424 CHD cases, 2,123 stroke cases, and 342 deaths from stroke. Characteristics of the included studies were presented in Table 1 for the analysis of CHD risk (12 studies) and Table 2 for the analysis of stroke risk (4 studies) and stroke-related mortality (2 studies). Of the 15 cohorts, 9 were conducted in European counties, 4 in the United States, and 1 in Japan, and 1 in Australia. The duration of follow-up ranged from 5 to 25 years. In addition to exclusion of participants with known pre-existing CHD and stroke, all studies also excluded those with diabetes at baseline except for the study by Mursu [13]. In the dietary assessment, 12 studies used validated food-frequency questionnaires, and the other 3 studies [13], [14], [17] used diet records or diet history interviews. Only the Nurses’ Health Study [10], [19], [23] updated dietary information during the follow-up and accounted for changes in dietary habits over time, whereas the others had only a single dietary measurement at baseline. Outcome assessments were from different sources including hospital discharge registries, death certificates, and medical records. All primary studies adjusted for age, BMI, smoking, physical activity, alcohol consumption, cereal fiber, and total energy intake. The multivariate adjusted RRs and 95%CI for CHD (Table S1), stroke (Table S2), and stroke-related mortality (Table S3) in the original articles were summarized.

**Table 1:** Characteristics of included prospective studies in this meta-analysis for dietary GI, GL and risk of CHD.

Study	Country	Studydesign	Samplesize	Age, yearsmean (SD)	Men	Mean BMI(kg/m^2^)	Follow-up(years)	Outcome(CHD)	Exposureassessment	Referencefood	Ascertainmentof Outcome	QualityScore*
Liu et al [10], 2000	United States	Cohort	75,521	38–63	0	24.8	10	761	Validated FFQ	White bread	Medical records, autopsy reports and death certificates	5
Van Dam et al [14], 2000	Netherlands	Cohort	646	71	646 (100%)	25.5	10	94	Cross-check dietary history method	White bread	Confirmed physician- administered or self-administered medical questionnaire, hospital discharge data	3
Halton et al [19], 2006	United States	Cohort	82,802	56 (7)	0	25.4	20	1,994	Validated FFQ	White bread	Medical records	5
Beulens et al [11], 2007	Dutch	Cohort	15,714	49–70	0	25.9	9	556	Validated FFQ	Glucose	Hospital discharge diagnoses	4
Levitan et al [15], 2007	Sweden	Cohort	36,246	45–79	36,246 (100%)	25.6	5	1,324	Validated FFQ	White bread	Hospital discharge diagnoses	4
Hardy et al [21], 2010	United States	Cohort	11,673	54.0 (White) 53.1(African American)	5,055 (43.3%)	26.6 (White) 29.1(African American)	17	1,312	Validated FFQ	White bread	Discharge diagnoses, death certificates, next-to-kin interviews, physician-completed questionnaires	4
Levitan et al [16], 2010	Sweden	Cohort	36,234	48–83	0	25.0	9	1,138	Validated FFQ	White bread	Records from Swedish inpatient and cause of death registers	4
Sieri et al [12], 2010	Italy	Cohort	44,132	35–64 (men) 35–74 (women)	13,637 (30.9%)	26.6 (men) 25.7(women)	7.9	463	Validated FFQ	Glucose	Records from mortality and hospital discharge databases	4
Burger et al [22], 2011	Dutch	Cohort	19,608	43(11)(men) 42.1(11.3)(women)	8,855 (45.2%)	25.4 (men) 24.7(women)	11.9	881	Validated FFQ	Glucose	Discharge diagnosis	4
Grau et al [17], 2011	Denmark	Cohort	3,774	30–70	1,885 (49.9%)	25.0 (men) 24.0(women)	6–25	363	Diet records or diet history interviews	Glucose	National Register of Cause of Death and National Register of Patients	3
Mursu et al [13], 2011	Finland	Cohort	1,981	52.5(5.3)	1,981 (100%)	26.7	16.1	376	Diet records	White bread	Hospital discharge diagnosis	3
Pierucci et al [20], 2011	Italy	Nested case control	204	67.35(8.26)(cases) 66.19(8.5)(controls)	124 (60.8%)	27.1(cases) 26.2(controls)	5	68	Validated FFQ	White bread	Discharge hospital records	4

Abbreviation: GI, glycemic index; GL, glycemic load; FFQ, food frequency questionnaire; BMI, body mass index.

* Study’s quality assessment was performed by review of study design, including inclusion and exclusion criteria, assessment of exposure, assessment of outcome, control of confounding, and evidence of bias. Each of the 5 quality criteria was evaluated and scored on an integer scale (0 or 1, with 1 being better) and summed; quality scores from 0 to 3 were considered lower and 4 to 5 higher quality.

**Table 2:** Characteristics of the included studies in this meta-analysis for dietary GI, GL and risk of stroke and related mortality.

Study	Country	Studydesign	Samplesize	Age, yearsmean(SD)	Men	MeanBMI (kg/m^2^)	Follow-up,years	Outcome(stroke)	Exposureassessment	Referencefood	Ascertainmentof Outcome	QualityScore*
**Stroke risk**												
Oh et al [23], 2005	United States	Cohort	78,779	46 (7)	0	24.0	18	794	Validated FFQ	White bread	Medical records	5
Beulens et al [11], 2007	Dutch	Cohort	15,714	49–70	0	25.9	9	243	Validated FFQ	Glucose	Hospital dischargediagnoses	4
Levitan et al [15], 2007	Sweden	Cohort	36,246	45–79	36,246 (100%)	25.6	5	857	Validated FFQ	White bread	Hospital discharge	4
Burger et al [22], 2011	Dutch	Cohort	19,608	43.0 (11.0) (men) 42.1 (11.3) (women)	8,855 (45.2%)	25.4 (men) 24.7(women)	11.9	229	Validated FFQ	Glucose	Discharge diagnoses	4
**Stroke mortality**												
Kaushik et al [25], 2009†	Australia	Cohort	2,897	65.3	1,274 (44.0%)	26.2	13	95	Validated FFQ	Glucose	Death data linkage with the Australian national death index	4
Oba et al [24], 2010	Japan	Cohort	27,862	53.7(12.1) (men) 54.9(13.0) (women)	12,561 (45.1%)	22.5 (men) 22.0 (women)	7.2	247	Validated FFQ	Glucose	Death data from Ministry of Internal Affairs and Communication	4

Abbreviations: GI, glycemic index; GL, glycemic load; FFQ, Food frequency questionnaire.

* Study’s quality assessment was the same as the footnote of Table 1.

† Only dietary GI was available in the original article.

### Glycemic Index or Glycemic Load and CHD Risk

**Figure 2:**
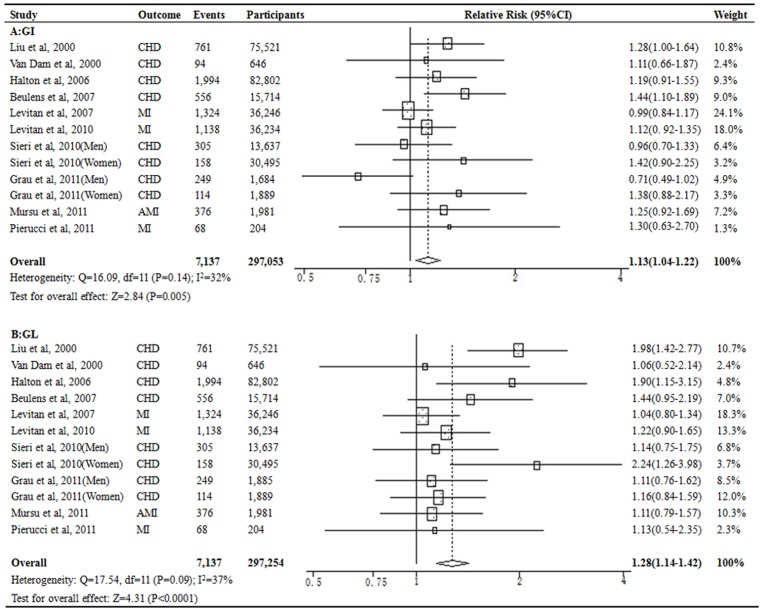
Relative risks for the association between dietary GI or GL and risk of CHD. The risk estimate and 95%CI were calculated by comparing the highest category with lowest.

A total of 12 separate estimates from 10 studies [10]–[17], [19], [20] were included in the analysis for the association between categories of GI and GL and CHD risk. Higher dietary GI levels were associated with a significant 13% increased risk for CHD (pooled RR 1.13, 95% CI, 1.04–1.22; *P* = 0.005) compared with the lowest category of dietary GI levels (Figure 2). There is no evidence of between-study heterogeneity (*I^2^* = 32%, *P* = 0.14). A pronounced association with CHD risk was observed for dietary GL. Compared with the lowest category of dietary GL levels, higher GL levels were associated with a significant 28% increased risk for CHD (pooled RR 1.28, 95% CI, 1.14–1.42; *P*<0.0001; Figure 2), with no heterogeneity between studies (*I^2^* = 37%; *P* = 0.09). Additional sensitivity analysis that excluded the study by Mursu et al [13] enrolling diabetic patients at baseline was conducted and the results did not change remarkably (pooled RR 1.12, 95%CI 1.03–1.21 for GI; pooled RR 1.30, 95%CI 1.15–1.46 for GL). Visual inspection of funnel plots did not identify important asymmetry (Figure S1), and no evidence of publication bias was observed by the Egger’s test (*P*>0.05).

**Figure 3:**
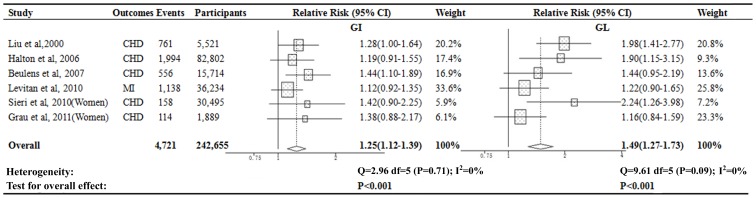
Relative risks for the association between dietary GI or GL and risk of CHD among women. The risk estimate and 95%CI were calculated by comparing the highest category with lowest.

When further stratified by gender, there is a gender-specific effect on the association of dietary GI and GL and the risk of CHD (*P* for interaction = 0.006 for GI; *P* for interaction = 0.005 for GL). A significant 49% increased risk of CHD for higher GL diet was observed in women (pooled RR = 1.49, 95%CI, 1.27–1.73; *P*<0.001; Figure 3), but not in men (pooled RR = 1.08; 95%CI, 0.91–1.27; *P* = 0.33) (Figure S2). Similarly, pooled RRs of CHD for higher GI diet were 1.25 (95%CI, 1.12–1.39; *P*<0.001) in women (Figure 3) and 0.99 (95%CI, 0.88–1.12; *P* = 0.90) in men (Figure S2).

We next assessed the potential effect modification by BMI on the relations of dietary GI and GL to CHD risk. For this analysis, results by BMI were available from 5 studies for dietary GI [11]–[13], [15], [16], and from 6 studies for dietary GL [10]–[13], [15], [16]. The cut-off point of BMI was 25 kg/m^2^ in 4 studies [11], [12], [15], [16], 27.5 kg/m^2^ in the Mursu study [13], 23 kg/m^2^ and 29 kg/m^2^ in the Liu study [10]. Because cut-off points of BMI varied across studies, we defined 2 subgroups as having a higher or lower BMI. In participants with a higher BMI, dietary GL and GI were associated with a significant increased risk of CHD; the pooled RRs were 1.49 (95%CI 1.27–1.76) for GL and 1.17 (95% CI 1.03–1.34) for GI, respectively. In those with a lower BMI, however, dietary GL or GI was not related to CHD risk (Table 3). Differences in pooled RRs by BMI reached statistical significance for GL (*P* for interaction = 0.003) but not for GI (*P* for interaction = 0.11).

**Table 3:** Stratified meta-analyses of association between dietary GI, GL and the risk of CHD by BMI.

		Dietary GI*						Dietary GL†					
Group		Data points	Pooled RR (95%CI)	*P*	‡ *P_interaction_*	§ *I^2^* (%)	§CochranQ test	Datapoints	Pooled RR (95%CI)	*P*	‡ *P_interaction_*	§ *I^2^* (%)	§Cochran Q test
Higher BMI	Overall	6	1.17 (1.03–1.34)	0.02	0.11	0	0.55	7	1.49 (1.27–1.76)	<0.001	0.003	59.8	0.02
	Women	3 [^11^], [12], [^16^]	1.24 (1.02–1.49)	0.03		0	0.87	4 [^10^], [11], [12], [^16^]	1.82 (1.44–2.31)	<0.001		0	0.51
	Men	3 [^12^], [13], [^15^]	1.12 (0.93–1.34)	0.25		37.1	0.20	3 [^12^], [13], [^15^]	1.28 (0.82–1.99)	0.28		73.0	0.02
Lower BMI	Overall	6	1.00 (0.86–1.16)	0.96		7.6	0.37	7	1.03 (0.86–1.23)	0.73		0	0.52
	Women	3 [^11^], [12], [^16^]	1.12 (0.92–1.36)	0.27		1.8	0.36	4 [^10^], [11], [12], [^16^]	1.17 (0.92–1.50)	0.20		0	0.42
	Men	3 [^12^], [13], [^15^]	0.87 (0.70–1.08)	0.20		0	0.77	3 [^12^], [13], [^15^]	0.89 (0.69–1.15)	0.39		0	1.00

* Analyses of dietary GI were based on 5 studies (6 data points, because men and women were included separately for the Beulens study [11]).

† Analyses of dietary GL were based on 6 studies (7 data points, because men and women were included separately for the Beulens study [11]).

‡ *P_interaction_* was for the difference in relative risks between higher and lower BMI overall.

§ The *I* ^2^ statistics and the Cochran Q test were used to examine statistical heterogeneity across studies.

### Glycemic Index or Glycemic Load and Stroke Risk

**Figure 4:**
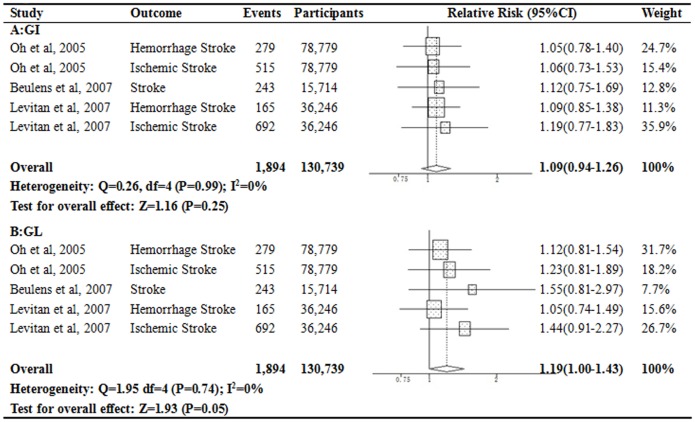
Relative risks for the association between dietary GI or GL and risk of stroke. The risk estimate and 95%CI were calculated by comparing the highest category with lowest.

A total of 5 separate estimates from 3 studies [11], [15], [23] were included in the analysis for the association between categories of GI and GL and stroke risk, comprising 130,739 participants and 1,894 incident stroke cases (Figure 4). There was no significant association between dietary GI and incident stroke, and pooled RR was 1.09 (95% CI, 0.94–1.26; *P* = 0.25) for the highest versus the lowest category of GI levels. High dietary GL level was associated with 19% increased risk for stroke (RR = 1.19; 95% CI, 1.00–1.43; *P* = 0.05). No evidence of heterogeneity across studies was observed.

### Glycemic Index and Stroke Mortality

A total of 3 separate estimates from 2 studies [24], [25] were included in the analysis for the association between category levels of GI and stroke mortality, comprising 30,759 participants and 342 deaths from stroke. We didn’t observe any significant association between stoke mortality and dietary GI (RR = 1.43; 95%CI, 0.98–2.09; *P* = 0.07; Figure S3), without observed between-study heterogeneity.

### Dose-relationship between Dietary GL, GI and Risk of CHD and Stroke

**Figure 5:**
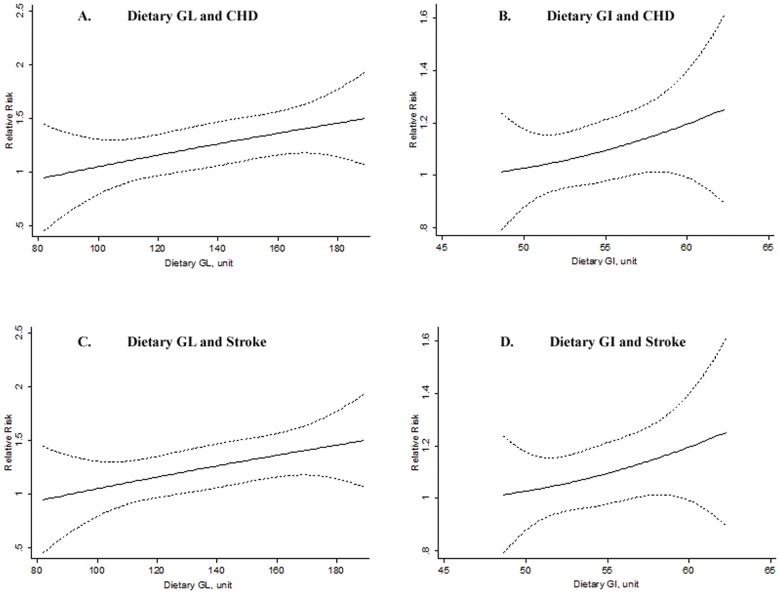
Dose-response relationship plot between GL, GI and risk of CHD and stroke. Dotted lines represent the 95% confidence intervals for the predicted relative risk. Dietary GL and GI values were converted to take glucose as the reference food. The dose-response relationship plot was conducted using the generalized least-squares trend estimation (GLST) analysis [33], based on available data for categories of dietary GL and GI on median dose, number of cases and participants, and effect estimates with corresponding standard errors. A: dietary GL and CHD risk (5 studies [10], [12], [13], [15], [16]); B: dietary GI and CHD risk (4 studies [12], [13], [15], [16]); C: dietary GL and stroke risk (2 studies [15], [23]); D: dietary GI and stroke risk (2 studies [15], [23]). The *P* values for nonlinear response test were 0.97 (A), 0.31 (B), 0.30 (C), and 0.42 (D).

**Figure 6:**
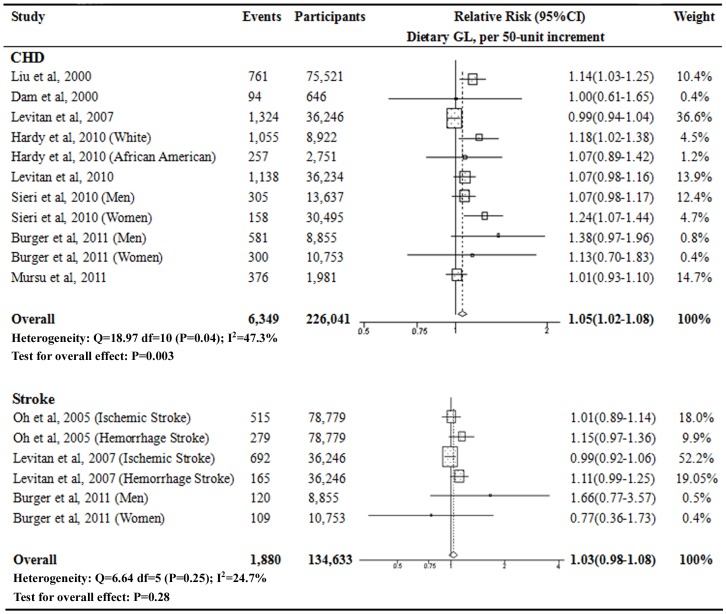
Relative risks of CHD and stroke by continuous dietary GL level. The 2-stage generalized least-squares trend estimation (GLST) method [33] was used to evaluate the relative risks of CHD and stroke by continuous dietary GL level, which allowed combining the GLST-estimated study-specific slopes with the results from studies that only reported effect estimates for continuous associations. The per 50-unit increment in dietary GI level was approximately equivalent to the difference between the medians of the highest and the lowest categories of the included studies.

The dose-response relationship plot between dietary GL, GI and the risk of CHD and stroke was estimated based on available data using the GLST meta-regression [33]. For CHD risk, a linear dose-response relationship was observed for dietary GL (*P* = 0.97 for nonlinear response test), and for dietary GI (*P* = 0.31 for nonlinear response test; Figure 5). For stroke risk, a linear dose-response relationship was also observed for dietary GL and GI (Figure 5). In addition, studies that reported continuous results for dietary GL and GI levels [14], [21], [22] were included in the 2-stage GLST dose-response analysis. The pooled RRs were 1.05 (95% CI 1.02–1.08; *P* = 0.003) for CHD risk and 1.03 (95% CI 0.98–1.08; *P* = 0.28) for stroke risk in per 50-unit increment of dietary GL levels, respectively (Figure 6). This increment was approximately equivalent to the difference between the medians of the highest and the lowest categories of the included studies. No associations were observed between continuous dietary GI level and the risk of CHD and stroke (Figure S4).

## Discussion

The present meta-analysis has quantitatively assessed the associations of dietary GL and GI with risk of CHD, stroke, and stroke-related mortality. Our results showed that gender significantly modified the effects of dietary GL and GI on CHD risk, and high dietary GL and GI are positively associated with increased CHD risk in women but not in men. The harmful influence of high dietary GL is more evident in overweight and obese subjects. In addition, high GL level was associated with 19% increased risk for stroke, while high GI level was not associated with stroke and stroke-related mortality.

It has been recognized that diet plays a major role in decreasing risk of cardiovascular diseases. Dietary GI and dietary GL are used to evaluate the glycemic properties of the diet. The first findings were reported from the Nurses Health Study where high dietary GL was observed to be associated with the risk of CHD [10] and later with hemorrhagic stroke [23], and these associations were the most evident in overweight women in both studies. Later, similar findings for CVD risk have been reported in several [11], [13], [16], but not all studies [12], [14], [15]. This meta-analysis of 12 prospective cohort studies supported that high dietary GL and GI are significantly associated with increased risk of CHD in women but not in men. This gender difference may be explained by the evidence that high GL and GI diets induce a more unfavorable cardiovascular risk profile in women than in men, such as dyslipidemia [36] and poor glycemic control [37].

Stratified meta-analysis by BMI indicated that among overweight and obese subjects, body weight may serve as an effect modifier in the association of high dietary GL with increased risk of CHD. The increasing demand of insulin in response to a high glycemic diet may exacerbate insulin resistance and lipid dysfunction in subjects with higher BMI [38], thus leading to a higher risk for developing CHD. Because of the varied BMI cut-off points across studies, however, further researches are needed to confirm the influence by body weight. The best way to investigate the influence of covariates, such as gender and the patients’ weight, is to perform a meta-analysis with studies’ individual data.

Our systematic review showed that high dietary GL, but not dietary GI, was associated with increased risk of stroke. The harmful effects were more pronounced for GL than for GI, which is expected as GL describes both quality and quantity of carbohydrates while GI represents only quality. Dietary GL is likely to be associated with more infusion of circulating glucose and higher postprandial insulin levels. One concern is that the relationship between GI or GL and stroke risk may be somewhat attenuated by combining ischemic stroke and hemorrhagic stroke in our analysis, because of the distinct pathogenesis of the 2 subtypes. High GI and GL diets can lead to endothelial impairment and vessel dysfunction mediated by the formation of advanced glycation end products, glycemia-induced oxidative stress, and inflammation [39], [40], and these changes may contribute to higher risk of stroke. Although ischemic stroke and hemorrhagic stroke also share common risk factors, such as hypertension, dyslipidemia, and atherosclerosis, large prospective cohort studies are needed to better understand the possible different effects of dietary GI and GL on risk of stroke and subtypes.

Several limitations should be considered carefully in the present meta-analysis. First, as in any observational study, our results could be influenced by differences in other factors. The diet patterns and dietary contributors to the GL vary in different populations. For example, white bread and potatoes are major contributors to the dietary GL in both the United States [41] and Sweden [42]. Cereal fiber intake such as crisp bread and whole-grain bread are substantially higher in the Swedish men than women in the Nurses’ Health Study [10], [41], [42]. While in Asian populations, white rice is the major contributor to the dietary GL, but with a low intake of fiber [24]. Second, because the exposure levels of the highest and lowest categories varied between studies, this difference may obscure the associations; nevertheless, our additional analysis that changed the exposures as continuous variables showed a consistent dose-response relationship between dietary GL and the risk of CHD. Among the included studies, only the Nurses’ Health Study had repeated dietary assessment during the follow-up period [10], while the others had a single dietary measurement. Misclassification of exposure to dietary GI and GL due to errors in completing the food-frequency questionnaire or changes in diet habits may have obscured the associations.

Third, even when conducted thoroughly, systematic reviews and meta-analysis are not immune to bias, including publication bias, small-study effect, and between-study heterogeneity. Some novel methods [43]–[45] have been developed to avoid the correlation between the natural log of odds ratio (InOR) or relative risk (InRR) and its standard error (and hence false-positive test results); however, most assessments of potential publication bias are indirect, rely on some assumptions, and usually require a large number of studies (at least 30 for sufficient power). In addition, between-study heterogeneity can lead to funnel plot asymmetry. There are several sources of the potential heterogeneity across studies, including poor methodological quality in study design, execution or analysis, and small studies targeting at high risk groups for whom the intervention may be most beneficial. In our meta-analysis, the test of heterogeneity using the Cochran Q test and the *I* ^2^ statistics showed no significant between-study heterogeneity, and there is little evidence of the publication bias as suggested by the Egger’s test. Nevertheless, even though the tests for publication bias are not significant, it is still very likely that negative studies are under published. Study registries with detailed knowledge of which studies have been published and which are unpublished would then be necessary to test publication bias accurately.

Finally, the use of dietary GI and GL is criticized for limited applicability in nutritional counseling and in the selection of foods to prevent and treat cardiovascular diseases. However, nutrition guidelines in western countries such as United States and Australia have currently recommended labeling foods with a symbol of their GI value, suggesting that it is applicable in public health recommendations.

In summary, our meta-analysis of all relevant prospective studies indicates that high dietary GI and GL are associated with increased risk of CHD in women but not in men, and the association was more pronounced between dietary GL and CHD, particularly in the overweight and obese subjects. High dietary GL was associated with increased risk of stroke. Clinical trials that aimed to evaluate the effect of reducing dietary GI or GL on the development of cardiovascular events should be performed in specific population.

## Supporting Information

Figure S1 Funnel plot of relative risk of dietary GI, GL and risk of CHD. Abbreviations: GI, glycemic index; GL, glycemic load; CHD, coronary heart disease.
(TIF)

Figure S2 Relative risks for the association between dietary GI or GL and risk of CHD in men. All the risk estimates and 95% CI were calculated by comparing the highest category with the lowest.
(TIF)

Figure S3 Relative risks for the associateion between dietary GI and stroke-related mortality. All the risk estimates and 95% CI were calculated by comparing the highest category with the lowest.
(TIF)

Figure S4 Relative risks of CHD and stroke by continuous dietary GI levels. The 2-stage generalized least-squares trend estimation (GLST) method [33] was used to evaluate the relative risks of CHD and stroke by continuous dietary GL level, which allowed combining the GLST-estimated study-specific slopes with the results from studies that only reported effect estimates for continuous associations. The per 10-unit increment in dietary GI level was approximately equivalent to the difference between the medians of the highest and the lowest categories of the included studies.
(TIF)

Table S1 Multi-variable adjusted RRs and 95%CI for CHD in the original articles in this meta-analysis.
(PDF)

Table S2 Multi-variable adjusted RRs and 95%CI for stroke in the original articles in this meta-analysis.
(PDF)

Table S3 Multi-variable adjusted RRs and 95%CI for stroke-related mortality in the original articles in this meta-analysis.
(PDF)
