# Supplementary figures and images for: Dietary Glycemic Index, Glycemic Load, and Risk of Coronary Heart Disease, Stroke, and Stroke Mortality: A Systematic Review with Meta-Analysis
Source: PLoS One. 2012 Dec 20;7(12):e52182. doi: 10.1371/journal.pone.0052182 (PMC3527433; doi:10.1371/journal.pone.0052182)

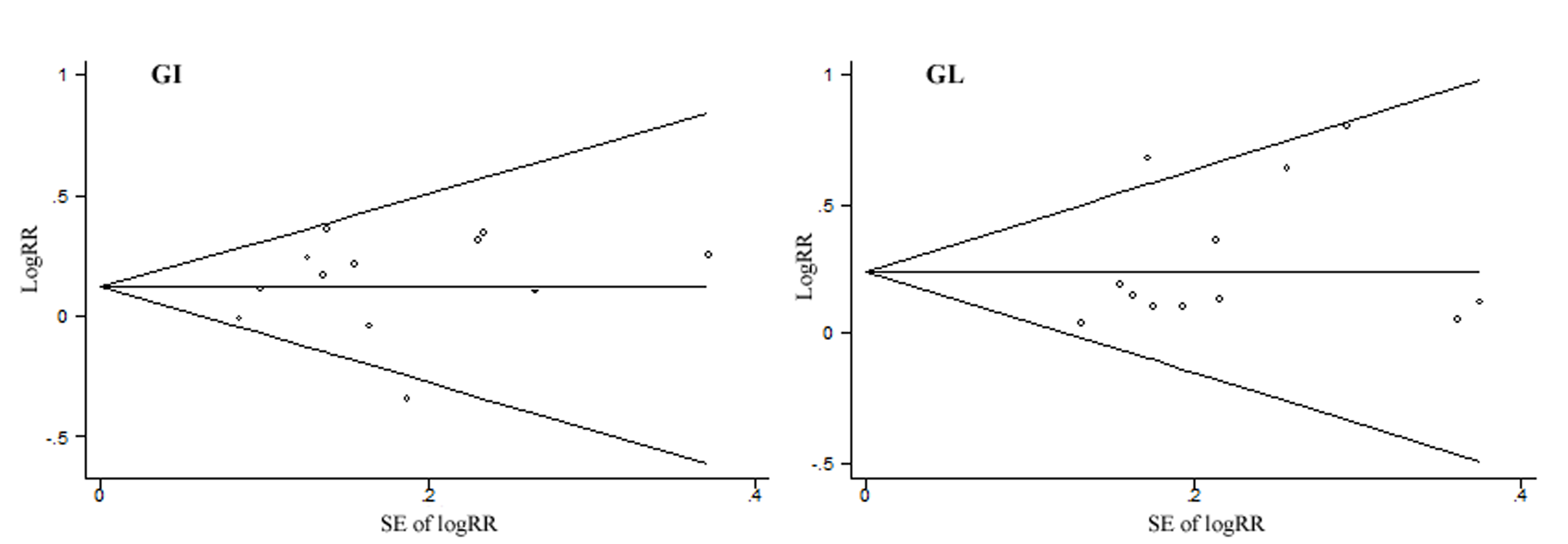

Supplement: Figure S1 — Funnel plot of relative risk of dietary GI, GL and risk of CHD. Abbreviations: GI, glycemic index; GL, glycemic load; CHD, coronary heart disease. (TIF) [file pone.0052182.s001.tif]

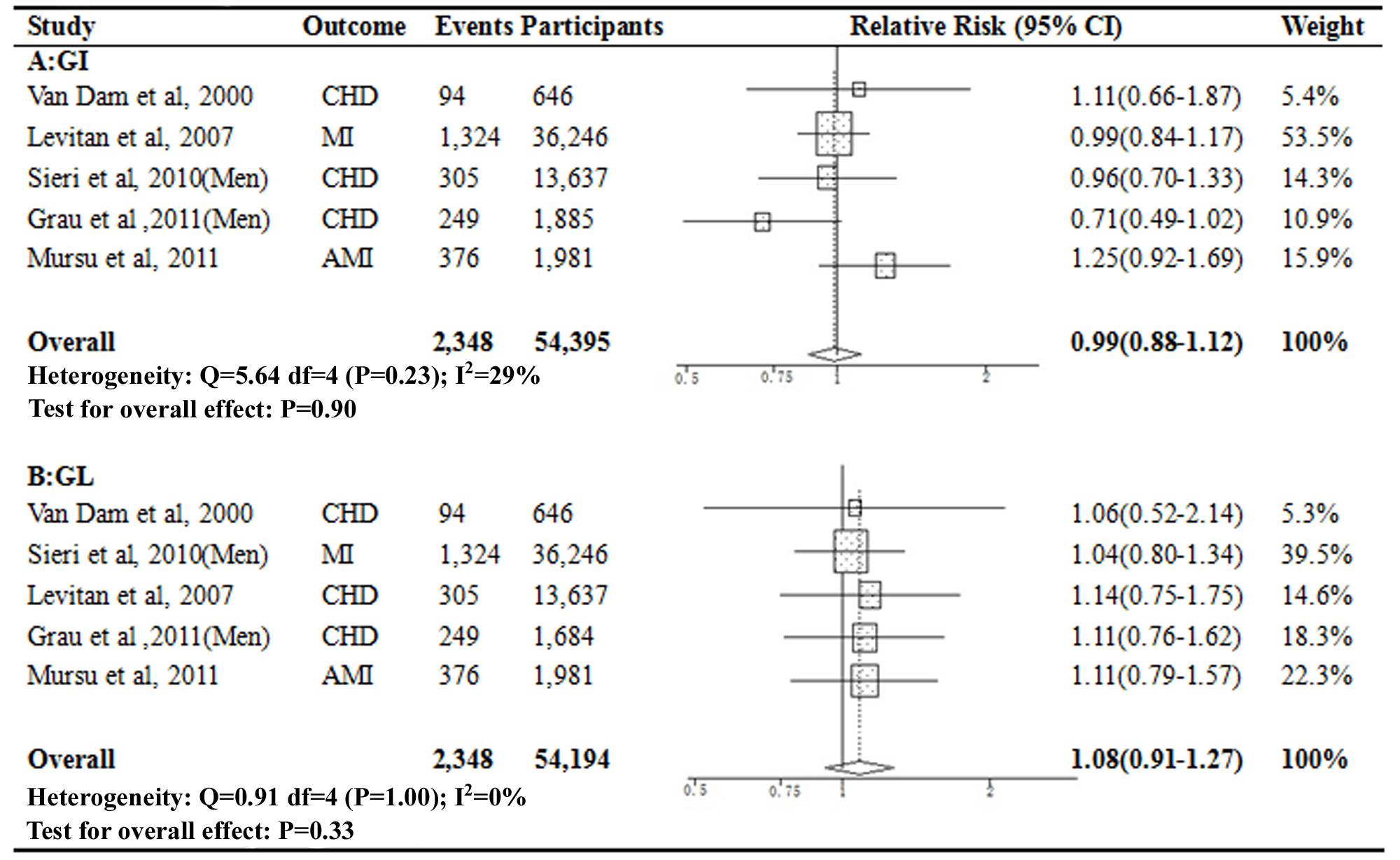

Supplement: Figure S2 — Relative risks for the association between dietary GI or GL and risk of CHD in men. All the risk estimates and 95% CI were calculated by comparing the highest category with the lowest. (TIF) [file pone.0052182.s002.tif]

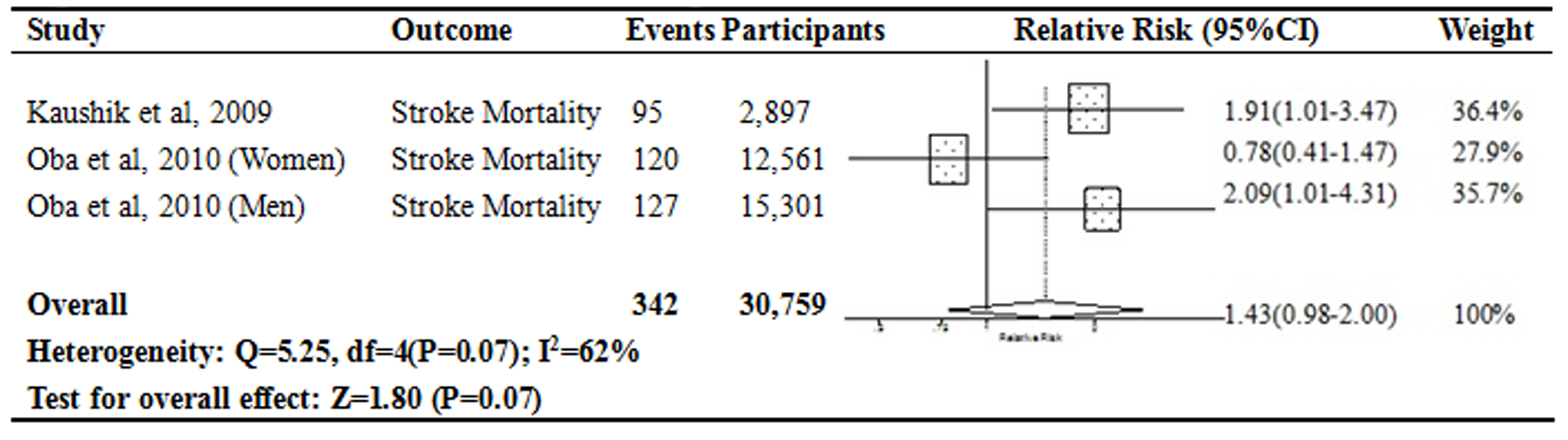

Supplement: Figure S3 — Relative risks for the associateion between dietary GI and stroke-related mortality. All the risk estimates and 95% CI were calculated by comparing the highest category with the lowest. (TIF) [file pone.0052182.s003.tif]

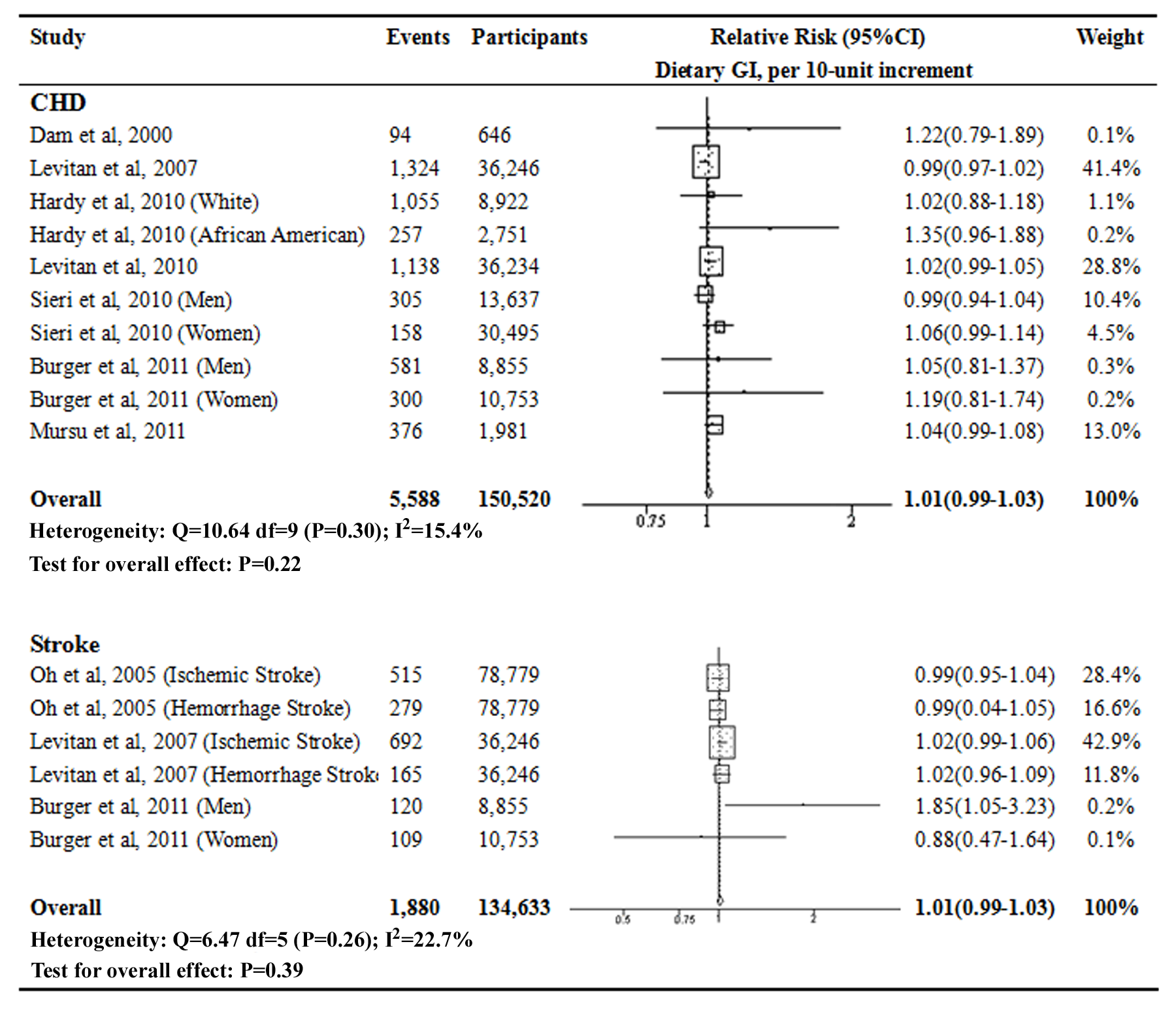

Supplement: Figure S4 — Relative risks of CHD and stroke by continuous dietary GI levels. The 2-stage generalized least-squares trend estimation (GLST) method [33] was used to evaluate the relative risks of CHD and stroke by continuous dietary GL level, which allowed combining the GLST-estimated study-specific slopes with the results from studies that only reported effect estimates for continuous associations. The per 10-unit increment in dietary GI level was approximately equivalent to the difference between the medians of the highest and the lowest categories of the included studies. (TIF) [file pone.0052182.s004.tif]
