# Supplementary material for: Dietary Glycemic Index, Glycemic Load, and Risk of Coronary Heart Disease, Stroke, and Stroke Mortality: A Systematic Review with Meta-Analysis
Source: PLoS One. 2012 Dec 20;7(12):e52182. doi: 10.1371/journal.pone.0052182 (PMC3527433; doi:10.1371/journal.pone.0052182)
Supplement: Table S1 — Multi-variable adjusted RRs and 95%CI for CHD in the original articles in this meta-analysis. (PDF) [file pone.0052182.s005.pdf]

Table S1. Multi-variable adjusted RRs and 95%CI for CHD in the original articles in this meta-analysis

Source	Comparison	Model	RR(95%CI)	Adjustment for Covariates
**Dietary GI**				
Liu et al, 2000 (Women)	Highest quintile *versus* lowest	Multivariate	1.28 (1.00-1.64)	Age; BMI; cigarette smoking; alcohol intake; parental family history of MI before the age of 60; self-reported history of hypertension or history of high cholesterol; menopausal status; aspirin use; use of multiple vitamin E supplement; physical activity; protein intake; intake of saturated, polyunsaturated, trans fats; dietary fiber intake; dietary vitamin E and folate intakes; total energy intake; and total carbohydrate intake.
Van Dam et al, 2000 (Men)	Highest tertile (median, 85) *versus* lowest (median, 77)	Crude<br>Multivariate	1.19 (0.72-1.96)<br>1.11 (0.66-1.87)	 Age; BMI; physical activity; smoking; prescribed diet; energy intake; intake of saturated fat, polyunsaturated fat, carbohydrates and alcohol; insulin; glucose; HDL-cholesterol; and log-transformed triacylglycerol concentration.
	Per 1-point increment	Multivariate	1.02 (0.98-1.07)	The same covariates mentioned above.
Halton et al, 2006 (Women)	Highest decile *versus* lowest	Multivariate	1.19 (0.91-1.55)	Age; BMI; smoking status; postmenopausal hormone use; hours of physical activity per week; alcohol intake; number of times aspirin was used per week; use of multivitamins; use of vitamin E supplement; history of hypertension; history of hypercholesterolemia; and parental history of MI.
Beulens et al, 2007 (Women)	Highest quartile *versus* lowest	Multivariate	1.44 (1.10-1.89)	Age; hypertension; cholesterolemia; smoking; BMI; mean SBP; total physical activity; menopausal status; hormone replacement therapy; oral contraceptives; alcohol intake; total energy intake; energy-adjusted intake of vitamin E, protein, dietary fiber, saturated fat, poly- and monounsaturated fat.
Levitan et al, 2007 (Men)	Highest quartile (median, 82.9) *versus* lowest (median, 73.0)	Model 1<br>Multivariate	1.12 (0.97-1.30)<br>0.99 (0.84-1.17)	Age<br>Age; BMI; physical activity; self-reported history of hypertension; family history MI before 60y of age; use of aspirin; cigarette smoking; marital status; education; and quartiles of intake of total energy, carbohydrate, saturated fat, polyunsaturated fat, alcohol, and cereal fiber.

Continuous Table S1

Source	Comparison	Model	RR (95%CI)	Adjustment for Covariates
Levitan et al, 2010 (Women)	Highest quartile (mean, 80.5) *versus* lowest (mean, 68.6)	Model 1<br>Multivariate	1.16 (0.98-1.37)<br>1.12 (0.92-1.35)	Age<br>Additional adjustment for education; BMI; physical activity; cigarette smoking; living alone; postmenopausal hormone use; aspirin use; total energy intake; alcohol intake; fiber intake; saturated fat; polyunsaturated fat; protein; carbohydrate; family history of MI before 60 years; self-reported history of hypertension; and self-reported history of high cholesterol
Sieri et al, 2010 (Men)	Highest quartile (mean, 56.9) *versus* lowest (mean 50.0)	Model 1<br>Multivariate	1.00 (0.73-1.37)<br>0.96 (0.70-1.33)	Age<br>Age; nonalcohol energy intake; hypertension; smoking; education; BMI; fiber intake; physical activity; and saturated fat intake.
Sieri et al, 2010 (Women)	Highest quartile (mean, 57.1) *versus* lowest (mean 49.9)	Model 1<br>Multivariate	1.47 (0.93-2.31)<br>1.42 (0.90-2.25)	Age<br>Age; nonalcohol energy intake; hypertension; smoking; education; BMI; fiber intake; physical activity; and saturated fat intake.
Grau et al, 2011 (Men)	95 percentile *versus* 50 percentile	Model 1<br>Multivariate	0.82 (0.58-1.17)<br>0.71 (0.49-1.02)	Age and total energy intake<br>Age; total energy intake; BMI; energy-adjusted carbohydrate, fat, protein and fiber intake; cohort; level of education; level of physical activity and smoking status; alcohol; saturated fat.
Grau et al, 2011 (Women)	95 percentile *versus* 50 percentile	Multivariate	1.38 (0.88-2.17)	The same covariates mentioned above.
Mursu et al, 2011 (Men)	Highest quartile *versus* lowest	Model 1<br>Multivariate	1.15 (0.86-1.53)<br>1.25 (0.92-1.69)	Age; examination years; and smoking<br>Age; examination years; smoking; BMI; SBP; hypertension medication; serum HDL and LDL-C; tryglycerides; leisure-time physical activity; education; family history of CVD; diabetes; alcohol; energy intake and energy adjusted intake of folate, fiber, vitamin C, polyunsaturated and saturated fat.
Hardy et al, 2010 (Whites)	Per 5-unit increment	Multivariate	1.01 (0.94-1.09)	Age; sex; BMI; SBP; total cholesterol; HDL-C; anti-hypertension medications; cigarette smoking; sports activity index; keys dietary score(calculated using polyunsaturated fat, dietary cholesterol and energy); fasting blood glucose; and total energy-adjusted GL.
Hardy et al, 2010 (Africa Americans)	Per 5-unit increment	Multivariate	1.16 (0.98-1.37)	The same covariates mentioned above.

Continuous Table S1

Source	Comparison	Model	RR(95%CI)	Adjustment for Covariates
Burger et al, 2011 (Men)	Per SD increment (SD=3.9)	Multivariate	1.02 (0.92-1.13)	Age; smoking; packyears; education; BMI; physical activity; hypertension; total energy; energy-adjusted carbohydrate and protein intake, alcohol, vitamin C, dietary fiber, saturated, monounsaturated and polyunsaturated fat, plasma TC, and HDL-C.
Burger et al, 2011 (Women)	Per SD increment (SD=3.9)	Multivariate	1.07 (0.92-1.24)	Age; smoking; packyears; education; BMI; physical activity; hypertension; oral contraceptives; total energy; energy-adjusted carbohydrate and protein intake, alcohol, vitamin C, dietary fiber, saturated, monounsaturated and polyunsaturated fat, plasma TC, and HDL-C.
Pierucci et al, 2011	Highest tertile *versus* lowest	Multivariate	1.30 (0.63-2.70)	Age; gender; BMI; cigarette smoking; hypertension.
**Dietary GL**				
Liu et al, 2000 (Women)	Highest quintile (Mean, 206) *versus* lowest (Mean, 117)	Model 1<br>Multivariate	1.57 (1.27-1.95)<br>1.98 (1.41-2.77)	Age and smoking<br>Additional adjustment for BMI;alcohol intake; parental family history of MI before the age of 60; self-reported history of hypertension or history of high cholesterol; menopausal status; aspirin use; use of multiple vitamin E supplement; physical activity; protein intake; dietary fiber, vitamin E, and folate intakes; and total energy intake; all fats.
Van Dam et al, 2000 (Men)	Highest tertile *versus* lowest	Multivariate	1.06 (0.52-2.14)	Age; BMI; physical activity; smoking; prescribed diet; energy intake; intake of saturated fat, polyunsaturated fat, and alcohol; insulin; glucose; HDL-cholesterol; log-transformed triylglycerol concentrations.
	Per 1-point increment	Multivariate	1.00 (0.99-1.01)	The same covariates mentioned above.
Halton et al, 2006 (Women)	Highest decile *versus* lowest decile	Model 1<br>Multivariate	1.13 (0.90-1.43)<br>1.90 (1.15-3.15)	Age and smoking<br>Additional adjustment for BMI; postmenopausal hormone use; hours of physical activity per week; alcohol intake; number of times aspirin was used per week; use of multivitamins; use of vitamin E supplement; history of hypertension; history of hypercholesterolemia; and parental history of MI; total protein; cereal fiber; saturated fat; polyunsaturated fat; monounsaturated fat; trans fat; and total calories.

Continuous Table S1

Source	Comparison	Model	RR (95%CI)	Adjustment for Covariates
Beulens et al, 2007 (Women)	Highest quartile (mean, 121.8) *versus* lowest (mean, 78.5)	Multivariate	1.44 (0.95-2.19)	Age; hypertension; cholesterolemia; smoking; BMI; mean SBP; total physical activity; menopausal status; hormone replacement therapy; oral contraceptives; alcohol intake; total energy intake; energy-adjusted intake of vitamin E, protein, dietary fiber, saturated fat, poly- and monounsaturated fat.
Levitan et al, 2007 (Men)	Highest quartile (median, 250) *versus* lowest (median, 180)	Model 1<br>Multivariate	1.03 (0.89-1.19)<br>1.04 (0.80-1.34)	Age<br>Additional adjustment for BMI; physical activity; self-reported history of hypertension; family history MI before 60y of age; use of aspirin; cigarette smoking; marital status; education; and quartiles of intake of total energy, carbohydrate, saturated fat, polyunsaturated fat, alcohol, and cereal fiber.
Levitan et al, 2010 (Women)	Highest quartile (mean, 188) *versus* lowest (mean, 128)	Model 1<br>Multivariate	1.09 (0.92-1.29)<br>1.22 (0.90-1.65)	Age<br>Additional adjustment for education; BMI; physical activity; cigarette smoking; living alone; postmenopausal hormone use; aspirin use; total energy intake; alcohol intake; fiber intake; saturated fat; polyunsaturated fat; protein; carbohydrate; family history of MI before 60 years; self-reported history of hypertension; and self-reported history of high cholesterol.
Sieri et al, 2010 (Men)	Highest quartile (mean, 189.0) *versus* lowest (mean 118.0)	Model 1<br>Multivariate	0.97 (0.70-1.34)<br>1.14 (0.75-1.75)	Age<br>Age; nonalcohol energy intake; hypertension; smoking; education; BMI; fiber intake; physical activity; and saturated fat intake.
Sieri et al, 2010 (Women)	Highest quartile (mean, 185.8) *versus* lowest (mean 122.8)	Model 1<br>Multivariate	1.97 (1.17-3.32)<br>2.24 (1.26-3.98)	Age<br>Additional adjustment for nonalcohol energy intake; hypertension; smoking; education; BMI; fiber intake; physical activity; and saturated fat intake.
Grau et al, 2011 (Men)	95 percentile *versus* 50 percentile	Multivariate	1.11 (0.76-1.62)	Age; total energy intake; BMI; cohort; level of education; level of physical activity.
Grau et al, 2011 (Women)	95 percentile *versus* 50 percentile	Multivariate	1.16 (0.84-1.59)	The same covariates mentioned above.
Pierucci et al, 2011	Highest tertile *versus* lowest	Multivariate	1.13 (0.54-2.35)	Age; gender; BMI; cigarette smoking; hypertension.

Continuous Table S1

Source	Comparison	Model	RR (95%CI)	Adjustment for Covariates
Mursu et al, 2011	Highest quartile (mean, 179.7) *versus* lowest (mean, 102.3)	Model 1<br>Multivariate	0.95 (0.71-1.26)<br>1.11 (0.79-1.57)	Age; examination years; smoking.<br>Additional adjustment for BMI; SBP; hypertension medication; serum HDL-C and LDL-C; triglycerides; leisure-time physical activity; education; family history of CVD; diabetes; alcohol; energy intake and energy adjusted intake of folate, fiber, vitamin C, polyunsaturated and saturated fat.
Hardy et al, 2010 (Whites)	Per 30-unit increment	Multivariate	1.11 (1.01-1.21)	Age; sex; BMI; SBP; total cholesterol; HDL-C; anti-hypertension medications; cigarette smoking; sports activity index; keys dietary score (calculated using polyunsaturated fat, dietary cholesterol and energy); fasting blood glucose; and total energy-adjusted GL.
Hardy et al, 2010 (Africa Americans)	Per 30-unit increment	Multivariate	1.04 (0.87-1.23)	The same covariates mentioned above.
Burger et al, 2011 (Men)	Per SD increment (SD=20.5)	Multivariate	1.14 (0.99-1.32)	Age; smoking; packyears; education; BMI; physical activity; hypertension; total energy; energy-adjusted alcohol, vitamin C, dietary fiber, and saturated, monounsaturated, and polyunsaturated fat, plasma TC, and HDL-C.
Burger et al, 2011 (Women)	Per SD increment (SD=20.5)	Multivariate	1.05 (0.86-1.28)	The same covariates mentioned above.

Abbreviations: GI, glycemic index; GL, glycemic load; BMI, body mass index; MI, myocardial infarction; SBP, systolic blood pressure; TC, total cholesterol; HDL-C, high-density lipoprotein cholesterol; RR, relative risk; CI, Confidence interval.
