# Supplementary material for: Dietary Glycemic Index, Glycemic Load, and Risk of Coronary Heart Disease, Stroke, and Stroke Mortality: A Systematic Review with Meta-Analysis
Source: PLoS One. 2012 Dec 20;7(12):e52182. doi: 10.1371/journal.pone.0052182 (PMC3527433; doi:10.1371/journal.pone.0052182)
Supplement: Table S2 — Multi-variable adjusted RRs and 95%CI for stroke in the original articles in this meta-analysis. (PDF) [file pone.0052182.s006.pdf]

Table S2. Multi-variable adjusted RRs and 95%CI for stroke in the original articles in this meta-analysis

Source	Comparison	Model	RR (95%CI)	Adjustment for Covariates
**Dietary GI**				
Oh et al, 2005 (Hemorrhagic stroke)	Highest quintile (median, 80.3) *versus* lowest (median, 68.0)	Model 1<br>Multivariate [a]	1.01 (0.71-1.44)<br>1.06 (0.73-1.53)	Age, smoking<br>Additional adjustment for BMI; alcohol intake; parental history for MI; history of hypertension, hypercholesterolemia, and diabetes; menopausal status and postmenopausal hormone use; aspirin use; multivitamin use; Vitamin E supplement use; physical activity; energy; cereal fiber.
Oh et al, 2005 (Ischemic stroke)	Highest quintile (median, 80.3) *versus* lowest (median, 68.0)	Model 1<br>Multivariate [a]	1.12 (0.85-1.48)<br>1.05 (0.78-1.40)	Age, smoking<br>Additional adjustment for BMI; alcohol intake; parental history for MI; history of hypertension, hypercholesterolemia, and diabetes; menopausal status and postmenopausal hormone use; aspirin use; multivitamin use; Vitamin E supplement use; physical activity; energy; cereal fiber.
Beulens et al, 2007	Highest quartile *versus* lowest	Multivariate [a]	1.12 (0.75-1.69)	Age; hypertension; cholesterolemia; smoking; BMI; mean SBP; total physical activity; menopausal status; HRT use; oral contraceptives; alcohol intake; total energy intake; energy-adjusted intake of vitamin E, protein, dietary fiber, saturated fat, poly- and monounsaturated fat.
Levitan et al, 2007 (Hemorrhagic stroke)	Highest quartile (median, 82.9) *versus* lowest (median, 73.0)	Model 1<br>Multivariate [a]	1.17 (0.77-1.79)<br>1.19 (0.77-1.83)	Age<br>Adjusted for age; BMI; physical activity; self-reported history of hypertension; cigarette smoking; and quartiles of intake of total energy and cereal fiber.
Levitan et al, 2007 (Ischemic stroke)	Highest quartile (median, 82.9) *versus* lowest (median, 73.0)	Model 1<br>Multivariate [a]	1.12 (0.90-1.39)<br>1.09 (0.85-1.38)	Age<br>Adjusted for age; BMI; physical activity; self-reported history of hypertension; family history MI before 60y of age; use of aspirin; cigarette smoking; marital status; education; and quartiles of intake of total energy, carbohydrate, saturated fat, polyunsaturated fat, alcohol, and cereal fiber.

Continuous Table S2

Source	Comparison	Model	RR (95%CI)	Adjustment for Covariates
Burger et al, 2011 (Men)	Per SD increment (SD=3.9)	Multivariate	1.27 (1.02-1.58)	Age; smoking; packyears; education; BMI; physical activity; hypertension; total energy; energy-adjusted carbohydrate and protein intake; alcohol; vitamin C; dietary fiber; saturated, monounsaturated and polyunsaturated fat; plasma TC; HDL-C.
Burger et al, 2011 (Women)	Per SD increment (SD=3.9)	Multivariate	0.95 (0.75-1.22)	Age; smoking; packyears; education; BMI; physical activity; hypertension; total energy; energy-adjusted carbohydrate and protein intake; alcohol; vitamin C; dietary fiber; saturated, monounsaturated and polyunsaturated fat; plasma TC; HDL-C.
**Dietary GL**				
Oh et al, 2005 (Hemorrhagic stroke)	Highest quintile (median, 166.8) *versus* lowest (median, 96.4)	Model 1<br>Multivariate [a]	1.01 (0.68-1.50)<br>1.23 (0.81-1.89)	Age, smoking<br>Additional adjustment for BMI; alcohol intake; parental history for MI; history of hypertension, hypercholesterolemia, and diabetes; menopausal status and postmenopausal hormone use; aspirin use; multivitamin use; Vitamin E supplement use; physical activity; energy; cereal fiber.
Oh et al, 2005 (Ischemic stroke)	Highest quintile (median, 166.8) *versus* lowest (median, 96.4)	Model 1<br>Multivariate [a]	1.12 (0.84-1.49)<br>1.12 (0.81-1.54)	Age, smoking<br>Additional adjustment for BMI; alcohol intake; parental history for MI; history of hypertension, hypercholesterolemia, and diabetes; menopausal status and postmenopausal hormone use; aspirin use; multivitamin use; Vitamin E supplement use; physical activity; energy; cereal fiber.
Beulens et al, 2007	Highest quartile (mean, 121.8) *versus* lowest (mean, 78.5)	Multivariate [a]	1.55 (0.81-2.97)	Age; hypertension; cholesterolemia; smoking; BMI; mean SBP; total physical activity; menopausal status; hormone replacement therapy; oral contraceptives; alcohol intake; total energy intake; energy-adjusted intake of vitamin E, protein, dietary fiber, saturated fat, poly- and monounsaturated fat.
Levitan et al, 2007 (Hemorrhagic stroke)	Highest quartile (median, 250) *versus* lowest (median, 180)	Model 1<br>Multivariate [a]	1.23 (0.80-1.90)<br>1.44 (0.91-2.27)	Age<br>Adjusted for age; BMI; physical activity; self-reported history of hypertension; cigarette smoking; and quartiles of intake of total energy and cereal fiber.

Continuous Table S2

Source	Comparison	Model	RR (95%CI)	Adjustment for Covariates
Levitan et al, 2007 (Ischemic stroke)	Highest quartile (median, 250) *versus* lowest (median, 180)	Model 1<br>Multivariate [a]	1.05 (0.85-1.29)<br>1.05 (0.74-1.49)	Age<br>Adjusted for age; BMI; physical activity; self-reported history of hypertension; family history MI before 60y of age; use of aspirin; cigarette smoking; marital status; education; and quartiles of intake of total energy, carbohydrate, saturated fat, polyunsaturated fat, alcohol, and cereal fiber.
Burger et al, 2011 (Men)	Per SD increment (SD=20.5)	Multivariate	1.23 (0.90-1.69)	Age; smoking; packyears; education; BMI; physical activity; hypertension; total energy; energy-adjusted carbohydrate and protein intake; alcohol; vitamin C; dietary fiber; saturated, monounsaturated and polyunsaturated fat; plasma TC; HDL-C.
Burger et al, 2011 (Women)	Per SD increment (SD=20.5)	Multivariate	0.90 (0.65-1.26)	Age; smoking; packyears; education; BMI; physical activity; hypertension; total energy; energy-adjusted carbohydrate and protein intake; alcohol; vitamin C; dietary fiber; saturated, monounsaturated and polyunsaturated fat; plasma TC; HDL-C.

Abbreviations: GI, glycemic index; GL, glycemic load; BMI, body mass index; MI, myocardial infarction; SBP, systolic blood pressure; TC, total cholesterol; HDL-C, high-density lipoprotein cholesterol; RR, relative risk; CI, Confidence interval.
