# Supplementary material for: Dietary Glycemic Index, Glycemic Load, and Risk of Coronary Heart Disease, Stroke, and Stroke Mortality: A Systematic Review with Meta-Analysis
Source: PLoS One. 2012 Dec 20;7(12):e52182. doi: 10.1371/journal.pone.0052182 (PMC3527433; doi:10.1371/journal.pone.0052182)
Supplement: Table S3 — Multi-variable adjusted RRs and 95%CI for stroke-related mortality in the original articles in this meta-analysis. (PDF) [file pone.0052182.s007.pdf]

Table S3. Multi-variable adjusted RRs and 95%CI for stroke-related mortality in the original articles in this meta-analysis

Source	Comparison	Model	RR (95%CI)	Adjustment for Covariates
Kaushik et al, 2009	Highest tertile (Median, 60.6) *versus* lowest (Median, 52.4)	Multivariate [a]	1.91 (1.01-3.47)	Age; gender; SBP; DBP; antihypertensive medication use; BMI; smoking status; educational qualifications; fair or poor self-rated health; history of MI and stroke; and presence of diabetes.
Oba et al, 2010 (Men)	Highest quartile (Mean, 70.3) *versus* lowest (Mean, 58.0)	Model 1	0.82 (0.49-1.37)	Age
		Multivariate [a]	0.78 (0.41-1.47)	Additional adjustment for BMI; smoking status; physical activity; reported history of hypertension; education; and intake of total energy, alcohol dietary fiber, salt and total fat.
Oba et al, 2010 (Women)	Highest quartile (Mean, 70.0) *versus* lowest (Mean, 58.3)	Model 1	2.46 (1.30-4.63)	Age
		Multivariate [a]	2.09 (1.01-4.31)	Additional adjustment for BMI; smoking status; physical activity; reported history of hypertension; education; and intake of total energy, alcohol dietary fiber, salt and total fat.

Abbreviations: GI, glycemic index; BMI, body mass index; MI, myocardial infarction; SBP, systolic blood pressure; DBP, diastolic blood pressure; RR, relative risk; CI, confidence interval
